# Supplementary figures and images for: Morphological and physiological characterization of filamentous Lentzea aerocolonigenes: Comparison of biopellets by microscopy and flow cytometry
Source: PLoS One. 2020 Jun 3;15(6):e0234125. doi: 10.1371/journal.pone.0234125 (PMC7269266; doi:10.1371/journal.pone.0234125)

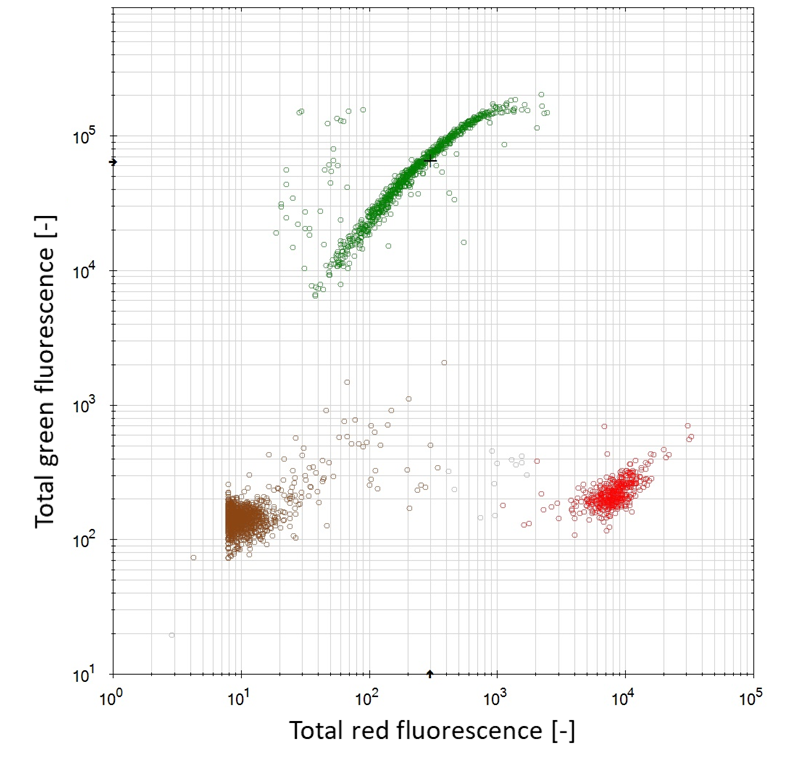

Supplement: S1 Fig — Populations of viable biomass (green), non-viable biomass (red) and background (brown) based on green (FDA positive) and red fluorescence (PI positive) signals. Total fluorescence signals represent the area underneath the curve with respect to flow cytometry signal profiles as depicted in Fig 6. (TIF) [file pone.0234125.s001.tif]

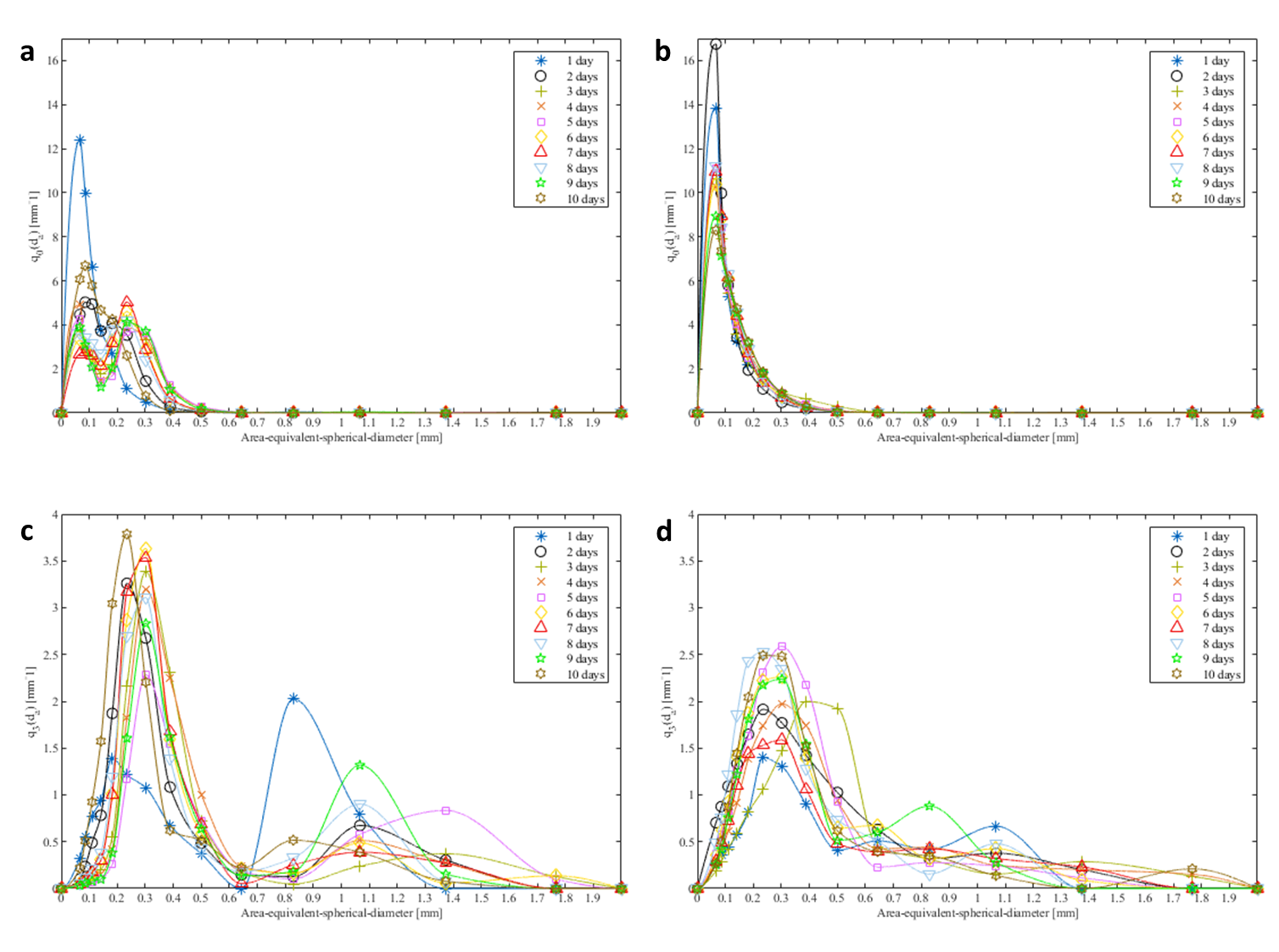

Supplement: S2 Fig — q0(da) of pellets from cultures (a) without and (b) with glass beads and q3(da) of pellets from cultures (c) without and (d) with glass beads. (TIF) [file pone.0234125.s002.tif]
