# Supplementary material for: Morphological and physiological characterization of filamentous Lentzea aerocolonigenes: Comparison of biopellets by microscopy and flow cytometry
Source: PLoS One. 2020 Jun 3;15(6):e0234125. doi: 10.1371/journal.pone.0234125 (PMC7269266; doi:10.1371/journal.pone.0234125)
Supplement: Attachment — Submitted filename: Response to Reviewers.docx [file pone.0234125.s004.docx]

Thank you very much for your letter with the reviewer assessment. In the name of all authors I would like to express our gratitude for the fast processing of our manuscript. We appreciate the comments on our paper. The suggestions given were processed. The revised manuscript was resubmitted as instructed.

Rainer Krull

| **Journal Requirements:** |  |
| --- | --- |
| 1. Please ensure that your manuscript meets PLOS ONE's style requirements, including those for file naming. | File naming is done according to the style requirements. |
| 2. We note that you have included the phrase “data not shown” in your manuscript. Unfortunately, this does not meet our data sharing requirements. PLOS does not permit references to inaccessible data. We require that authors provide all relevant data within the paper, Supporting Information files, or in an acceptable, public repository. Please add a citation to support this phrase or upload the data that corresponds with these findings to a stable repository (such as Figshare or Dryad) and provide and URLs, DOIs, or accession numbers that may be used to access these data. Or, if the data are not a core part of the research being presented in your study, we ask that you remove the phrase that refers to these data. | We agree and added the figures that confirm our statement in the supporting information,  (S2 Fig). |
| 3. Thank you for providing the following Funding Statement:  "The authors gratefully acknowledge financial support by the German Research Foundation (DFG) in the Priority Programme 1934 DiSPBiotech – Dispersity, structural and phase modifications of proteins and biological agglomerates in biotechnological processes (SPP 1934 DiSPBiotech – 315384307 (HB) and 315457657 (RK/AK)). The work of LV and CH was supported by the Austrian Research Promotion Agency (FFG) (Grant number: 844608) and within the framework of the Competence Center CHASE GmbH, funded by the Austrian Research Promotion Agency (grant number 868615) as part of the COMET program-Competence Centers for Excellent Technologies by BMVIT, BMDW, the Federal Provinces of Upper Austria and Vienna. The funders had no role in study design, data collection and analysis, decision to publish, or preparation of the manuscript."   We note that one or more of the authors is affiliated with the funding organization, indicating the funder may have had some role in the design, data collection, analysis or preparation of your manuscript for publication; in other words, the funder played an indirect role through the participation of the co-authors.  If the funding organization did not play a role in the study design, data collection and analysis, decision to publish, or preparation of the manuscript and only provided financial support in the form of authors' salaries and/or research materials, please review your statements relating to the author contributions, and ensure you have specifically and accurately indicated the role(s) that these authors had in your study in the Author Contributions section of the online submission form. Please make any necessary amendments directly within this section of the online submission form.  Please also update your Funding Statement to include the following statement: “The funder provided support in the form of salaries for authors [insert relevant initials], but did not have any additional role in the study design, data collection and analysis, decision to publish, or preparation of the manuscript. The specific roles of these authors are articulated in the ‘author contributions’ section.”   If the funding organization did have an additional role, please state and explain that role within your Funding Statement.   Please also provide an updated Competing Interests Statement declaring this commercial affiliation along with any other relevant declarations relating to employment, consultancy, patents, products in development, or marketed products, etc.   Within your Competing Interests Statement, please confirm that this commercial affiliation does not alter your adherence to all PLOS ONE policies on sharing data and materials by including the following statement: "This does not alter our adherence to  PLOS ONE policies on sharing data and materials.” (as detailed online in our guide for authors [http://journals.plos.org/plosone/s/competing-interests](about:blank)). If this adherence statement is not accurate and  there are restrictions on sharing of data and/or materials, please state these. Please note that we cannot proceed with consideration of your article until this information has been declared.  Please know it is PLOS ONE policy for corresponding authors to declare, on behalf of all authors, all potential competing interests for the purposes of transparency. PLOS defines a competing interest as anything that interferes with, or could reasonably be perceived as interfering with, the full and objective presentation, peer review, editorial decision-making, or publication of research or non-research articles submitted to one of the journals. Competing interests can be financial or non-financial, professional, or personal. Competing interests can arise in relationship to an organization or another person. Please follow this link to our website for more details on competing interests: [http://journals.plos.org/plosone/s/competing-interests](about:blank) | The funding statement was updatet:  The funder provided support in the form of salary for author LV, but did not have any additional role in the study design, data collection and analysis, decision to publish, or preparation of the manuscript. The specific roles of these authors are articulated in the ‘author contributions’ section.  Competing Interests:  The following remark is added in the Competing Interest Statement during resubmission:  Competence Center CHASE GmbH participated in the salary of LV. This does not alter our adherence to  PLOS ONE policies on sharing data and materials. |
| **Reviewer #1:** |  |
| General comments: Legends should be inserted close to the Figures, in order to make easy the reading of the paper. Figures are not presented sequentially; Figure 4 is not referred in the text. | We apologize for any confusion. Figure and legend placement were a demand of the journal. Final formatting will be done by PLOS ONE, Figures and legends will be placed close to each other.  Furthermore, figures are presented sequentially and are referred to in the text of the revised manuscript. |
| Line 116 – Authors should specify if glucose was separately sterilized | Specified in lines 119-120 |
| Line 177 – Authors referred that they have used Syto9 and PI dyes for CLSM analysis; however, they did not explain how these dyes work, as they did - and well done – for the Flow cytometry protocol, in which they explained how PI and FDA work (Line 221). | Explained in lines 194-198 |
| Line 223 – Please indicate the number of events/s, threshold and compensation settings for FC analysis; | We have added clarifying remarks in lines 231-240 |
| Line 276 – “The transition from growth phase to stationary phase occurs between day 1 and 2 (Fig 2b)”. This is wrong, since the growth phases are identified in natural logarithmic (Ln) biomass versus time plots, not in biomass versus time plots, as Fig. 2b) shows. Please correct the sentence. | Changed in lines 298-299 |
| Line 338 – “Due to the high manual effort of pellet slicing only one pellet per sample point was examined.” Do Authors think that this procedure is statistically relevant? Please comment. | The well-established method to determine pellet viability via CLSM was applied to gain qualitative insights. To investigate a statistically relevant number of pellets, flow cytometry has been applied (following section).  Explained in lines 361-362. |
| -It would be interesting to see the cytograms with FDA/PI results | We have included the respective cytogram. S1 Fig displays an exemplary flow-cytometry scatterplot. Clarifying comments can be found in line 226-229 as well as caption for S1 Fig. |
| Figure 2 a) –Some experimental points show an error > 20%, which is unacceptable; The same for the biomass curve depicted in Figure 2 b) More experimental points should be presented during the first 24 h, in order to have a more accurate biomass profile. | Working with filamentous microorganisms and their complex morphology is challenging, often leading to larger errors than expected for e.g. unicellular microorganisms. These relatively large errors are also found in many literature references cited in this work.  Experimental points are limited due to large volumes needed for analysis. Sampling every day was chosen to depict the whole process. In the first 24 hours, no rebeccamycin production was expected. Thus, we did not focus on the biomass profile during the first 24 hours. |
| Figures 3) and b) should present error bars. | We chose not to display error bars since pellet size distributions are shown. All pellets from microscopic images were considered in Fig 3 and the differences/errors are shown by the distribution. |
|  | |
| **Reviewer #2:** |  |
| I have read throughout the manuscript; it’s a valuable and comprehensive research showing metabolic activity in macro morphology of filamentous bacteria producing rebeccamycin. However, in several instances, the presentation and the discussion of the achieved results and the comparison with the relevant literature are rather limited. | We thank the reviewer very much for the motivating comment. The discussion was extended and comparisons with relevant literature were added (see corresponding section). |
| 1. Provide a suitable reference for sentence mentioned in line no 405 to 406 for metabolic activity. | Indeed, the sentence about metabolic activity is better suited to data from flow cytometry and the use of fluorescein diacetate. Consequently, we have revised this, see lines 437-441. |
| 2. Is there any evidence that rebeccamycin has fluorescence properties (Line no 427-429). | Yes, see lines 473-474. |
| 3. Any suitable experiment/reference showing that the smaller and uniform morphology supports more substrate uptake and oxygen into pellet. However, results are contradictory, where all glucose are utilized in 2 days under both condition (With glass beads and without glass beads. Explain this. | Suitable references were added, see lines 449-457.  We apologize for the confusion, in our estimation results are not really contradictory as only the facilitated transport of oxygen and not the consumption of glucose is described. Fungal pellets often have a 200 µm layer which is well supplied (Hille et al. 2005), so small pellets can be totally supplied while larger pellets show limitations in the center. |
| 4. Did you optimize the substrate concentration during experiment (>3.5 g/L). | 4 g/L of glucose were used in the beginning, no optimization was conducted. The glucose concentration was based on the medium composition used by Walisko et al. (2017) with glass bead addition. |
| 5. Why authors used only 100 g/L of beads or with same diameter, did they check if more or less quantity of beads have different observation than the current study? | In Schrader et al. (2019) different glass bead diameters at 100 g/L were investigated regarding their effects on final product titers. However, only the size of 969 µm showed a significant difference compared to an unsupplemented cultivation shown by statistical analysis. In order to test the different methods in this manuscript two different conditions that were known to be significantly different were chosen. Different concentrations of glass beads would of course affect the cultivation, but were not investigated in such detail as described in this manuscript. |
| 6. Please provide statistical analysis of data. | We agree with Reviewer 2 and have added a statistical analysis (S3 Table in supporting information as well as lines 278-284 and 418-424) |
